# GPM6B Inhibit PCa Proliferation by Blocking Prostate Cancer Cell Serotonin Absorptive Capacity

**DOI:** 10.1155/2020/8810756

**Published:** 2020-11-25

**Authors:** Siyuan He, Zhenlin Huang, Xiang Li, Yinghui Ding, Haoyue Sheng, Bowen Liu, Zhankui Jia

**Affiliations:** ^1^Department of Urology, The First Affiliated Hospital of Zhengzhou University, Zhengzhou, China 450000; ^2^Department of Urology, Fudan University Shanghai Cancer Center, Shanghai, China 200000

## Abstract

Prostate cancer is currently one of the most common fatal tumor types in men. Although multiple treatments can alleviate some cases, advanced prostate cancer, especially CRPC, still has a very poor prognosis. Therefore, early detection and diagnosis of prostate cancer have a very important role in the prognosis of patients. Glycoprotein M6B (GPM6B) is a transmembrane protein that belongs to the proteolipid protein family. GPM6B has been proved and can be used as a biomarker for gynecological malignancies and breast carcinoma. However, there are no studies that explored the functions of GPM6B in PCa. We explored differentially expressed genes in prostate cancer by analyzing TCGA data and found GPM6B downregulated in PCa tissues compared to that in normal prostate tissues. The GPM6B expression in PCa patient's tumor tissues was significantly related to clinical stage, T classification, lymph node metastasis, and distant metastasis, but not significantly related to age and Gleason score. Also, patients with highGPM6B expression had a better prognosis. The overexpression of GPM6B in prostate cancer cells could inhibit cell proliferation. Serotonin treatment could enhance the proliferation of PCa cell lines; moreover, fluoxetine could reverse this result. In conclusion, we identified GPM6B as a tumor suppressor in PCa. In mechanism, it can regulate the uptaking of serotonin and inhibit the growth of prostate cancer. These results suggested the potential function of GPM6B as a diagnostic marker of PCa and provided clues for the development of new treatment targets for PCa.

## 1. Introduction

Prostate cancer (PCa), the second most common cancer in men, is associated with high morbidity and mortality [1]. With changes in diet and ageing of the population, the number of patients diagnosed with PCa in recent years has gradually increased [1, 2]. The main cause of PCa-related death is still metastasis [2, 3]. However, traditional treatments are not very effective in treating castration-resistant metastasis [4, 5]. For this reason, exploring the molecular mechanisms that promote PCa progression could supply ideas for a valid therapeutic strategy.

Glycoprotein M6B (GPM6B) is a transmembrane protein that belongs to the proteolipid protein family. In addition, GPM6A and DM20 also belong to the proteolipid protein family [6]. Earlier studies have shown that proteolipid proteins might work as housekeeping proteins that participate in intracellular transport [7]. GPM6B is widely distributed among cells of the central nervous system, such as neurons and oligodendrocytes [8, 9]. In the nervous system, GPM6B is involved in the process of neuronal myelination, maintains the stability of axonal membranes, and promotes neuronal differentiation [10–12]. In addition, GPM6B plays a role in regulating osteoblast function and mineralization induction [13]. The main function of the serotonin transporter (SERT) is to transport serotonin. Relevant studies have shown that GPM6B can bind to SERT and result in a significant reduction in serotonin uptake [14]. GPM6B also plays a regulatory role in smooth muscle cell differentiation [15]. Oncological research on GPM6B has gradually attracted attention in recent years. In breast cancer, GPM6B can be used as a marker of tumor angiogenesis [16] In a study of the gynecological malignancies, namely, endometrial cancer, uterine cancer, and ovarian cancer, the expression level of Gpm6B was markedly elevated in patient tumor tissues and higher in the peripheral blood of patients than that of healthy controls [17]. Acting as a protooncogene, GPM6B also upregulates expression in a variety of types of human B lymphocytic leukemia [18]. Although some studies on GPM6B have been conducted, there has been no research on the GPM6B protein expression pattern and the relationships between GPM6B expression and clinical-pathological data in PCa. The aims of this study were as follows: (1) investigate the differential expression of GPM6B in PCa and adjacent normal tissues; (2) investigate the effect of GPM6B on the clinical prognosis of PCa patients; and (3) explore the function and mechanism of GPM6B in PCa.

## 2. Materials and Methods

### 2.1. Data Mining

For this study, we selected three Gene Expression Omnibus (GEO) datasets from PCa tissues and normal prostate tissues to analyze the expression pattern of GPM6B in PCa. The main data can be found in GEO datasets GSE46602, GSE69223, and GSE27616 (https://www.ncbi.nlm.nih.gov/geo). We compared PCa sample data from each GEO dataset with normal prostate tissue sample data to identify differentially expressed genes with the online program GE02R based on the limma R package (http://www.ncbi.nlm.nih.gov/geo/geo2r/). False-positive results were corrected by adjusting the *p* value. Therefore, we chose a “*p* value < 0.05” and a “logFC > 1.2” as the main selection criteria. Differentially expressed genes in all three datasets that satisfied these criteria were selected as significantly differentially expressed genes. To uncover the prognostic value of GPM6B in the Cancer Genome Atlas (TCGA) cohort, the Gene Expression Profiling Interactive Analysis (GEPIA) database (http://gepia.cancer-pku.cn/) was used. The Database for Annotation, Visualization, and Integrated Discovery (DAVID) database can provide functional annotation of numerous genes from a variety of genomic resources (https://david.ncifcrf.gov/). Gene Ontology (GO) and Kyoto Encyclopedia of Genes and Genomes (KEGG) pathway analyses were performed using the DAVID database.

### 2.2. Tissue Specimens

A total of 94 paraffin-embedded tissue specimens were collected from PCa patients treated at the First Affiliated Hospital of Zhengzhou University (Zhengzhou, China) from August 2011 to December 2013; of these specimens, 54 were surgical specimens, and 40 were biopsy specimens. The patients in the study cohort were all confirmed by pathological diagnosis and have not received chemotherapy. Written informed consent was signed by every participant who participated in this research. This study was reviewed by the Research Ethics Committee of the First Affiliated Hospital of Zhengzhou University (trial registration number: 2019-KY-236).

### 2.3. Immunohistochemistry (IHC)

Paraffin-embedded tissues were sliced into 5 *μ*m sections before immunohistochemistry. After deparaffinization and gradual rehydration, the tissue sections were placed in citrate buffer (G1202, Servicebio, China) and heated for antigen retrieval. After cooling, the slides were placed in phosphate-buffered saline (PBS) and shaken on a shaker three times. After endogenous peroxidase activity was blocked using a 3% hydrogen peroxide solution, the sections were washed 3 times in PBS on a shaker, and the tissues were then covered with 3% bovine serum albumin (BSA) (G5001, Servicebio, China) and incubated for 30 minutes. Then covered with diluted anti-GPM6B antibody (1 : 500, ab224318, Abcam, UK) and incubated for 24 hours at 4°C. Washed the sections 3 times in PBS, and the slides were covered with antibody (GB23303, Servicebio, China) and incubated for 50 minutes at room temperature. Finally, add diaminobenzidine (DAB) (K5007, DAKO, USA) after washing with PBS; the development time was controlled by monitoring under a microscope, and the sections were rinsed with water. Then, the cells were counterstained with hematoxylin for 3 minutes.

Staining scores were blindly and independently confirmed by two senior pathologists who assessed the positive cell frequency and the staining intensity. The positive cell frequency was defined as follows: 1, 1%–9% positive cells; 2, 10%–50%; 3, 51%–80%; and 4, >80% positive cells. The staining intensity was scored as follows: 0, negative staining; 1, weak staining; 2, moderate staining; and 3, strong staining. The immunoreactive score (IRS) was the product of the positive cell frequency and the staining intensity.

### 2.4. Quantitative Real-Time PCR (qPCR)

Total RNA was extracted using TRIzol (NR0002, Leagene, China). Then, total RNA was reverse transcribed into cDNA using ReverTra Ace qPCR RT Master Mix with gDNA Remover (TOYOBO, Japan). Gene transcripts were quantified on a QuantStudio 3 Real-Time PCR System (Thermo Fisher, USA) using the FastStart Essential DNA Green Master Mix (Roche, USA), and the expression was normalized to that of GAPDH. The raw data are presented as the relative expression level, which was calculated using the 2^-△△Ct^ method. The primers (Sangon Biotech, China) used for qPCR were listed as below: GPM6B: 5′-TGAGCGAGGTGATACAACTGATGC-3′ and 5′-GCCACTCCAAGCACATAGGTGAG-3′; GAPDH: 5′-CGGAGTCAACGGATTTGGTCGTAT-3′ and 5′-AGCCTTCTCCATGGTGGTGAAGAC-3′.

### 2.5. Development of a GPM6B Overexpression Plasmid

The GPM6B coding sequence (CDS) was first amplified by PCR. Then, we inserted the GPM6B CDS into the pHBLV-CMV-MCS-3FLAG-EF1-ZsGreen-T2A-PURO vector (Hanbio Biotechnology, China) using an HB-infusion™ Cloning Kit (Hanbio Biotechnology, China). The PCR primer sequences were 5′-TGAGCGAGGTGATACAACTGATGC-3′ and 5′-GCCACTCCAAGCACATAGGTGAG-3′. The GPM6B overexpression plasmid was validated by DNA sequencing. Amplification of the GPM6B plasmid was performed using DH5*α* competent cells (Thermo Fisher, USA).

### 2.6. Cell Culture and Transfection

The PCa cell lines DU145 and 22RV1 were purchased from the American Type Culture Collection (ATCC, USA). We cultured cell lines using minimum Eagle's medium (MEM) (HyClone, USA) and Roswell Park Memorial Institute- (RPMI-) 1640 medium (HyClone, USA) supplemented with 10% foetal bovine serum (Gibco, USA). The cells were placed in a cell culture incubator at 37°C with a humid atmosphere containing 5% CO_2_. Cells were transfected using Lipofectamine 3000 (Thermo Scientific, USA) following the manufacturer's specifications when they attained 50-60% confluence.

### 2.7. Western Blot

We used sodium dodecyl sulfate-polyacrylamide gel electrophoresis (SDS-PAGE) gels to separate the total protein (100 *μ*g), and proteins were then transferred onto nitrocellulose membranes (Millipore, USA). The blotted membranes were probed with anti-GPM6B antibody (1 : 1000, ab224318, Abcam, UK). After washing, the membranes were incubated with HRP-conjugated goat anti-rabbit IgG antibody (1 : 5000, AS014, ABclonal, China). The blots were developed using Immobilon Western Chemiluminescent HRP Substrate (Millipore, USA). Anti-*β*-actin antibody (1 : 1000, AC006, ABclonal, China) was used as a loading control. Then, we used an Amersham Imager 600 (GE, USA) to detect the bands.

### 2.8. Cell Counting Kit-8 (CCK8) Assay

Cell lines in the logarithmic growth phase were digested, counted, inoculated in 96-well plates at a density of 2500 cells per well, and cultured for 0, 1, 2, 3, 4, 5, and 6 days. Finally, the experimental results were detected using the CCK8 assay (Dojindo, Japan). The absorbance at 450 nm was measured using a DNM-9606 microplate reader (Perlong, China). Serotonin (Solarbio, China) and fluoxetine (Solarbio, China) were introduced during the experiment.

### 2.9. Colony Formation Assay

Cell lines in the logarithmic growth phase were digested and counted. 1000 cells were seeded in each dish. After culturing for 1 week, the colonies were stained with Giemsa stain. Then, the colonies were counted under the microscope.

### 2.10. Statistical Analyses

Statistical analyses were performed using SPSS 21.0 statistical software (SPSS, Chicago, IL, USA) or GraphPad Prism (GraphPad Software, Inc., San Diego, CA). The relationship between GPM6B expression and clinicopathological parameters was verified using the chi-square test. The survival probability was estimated using the Kaplan-Meier method, and the log-rank test was used to compare overall survival (OS) between groups. Univariate and multivariate Cox regression analyses were performed to identify factors with a significant influence on survival. Repeat all experiments at least three times, and the results shown were representative of these experiments. Differences at *p* < 0.05 were considered to be statistically significant.

## 3. Results

### 3.1. Low Expression of GPM6B Is Highly Correlated with PCa

**Figure 1:**
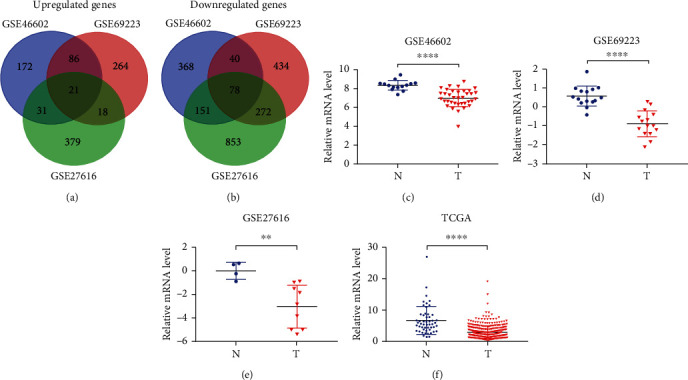
Low expression of GPM6B is highly correlated with PCa. (a) Upregulated differentially expressed genes in different prostate cancer GEO datasets. (b) Downregulated differentially expressed genes in different prostate cancer GEO datasets. (c–f) GPM6B expression was significantly decreased in PCa tissues compared to normal prostate tissues in different GEO datasets and a TCGA cohort.

We selected three gene expression datasets (GSE46602, GSE69223, and GSE27616) from the GEO database to identify the most significant differential genes. The compositions of the GEO datasets were shown in Table 1. Then, we compared gene expression in PCa tissues with normal prostate tissues using an online program GEO2R. Here, we identified 971 upregulated differentially expressed genes (Figure 1(a)) and 2196 downregulated differentially expressed genes (Figure 1(b)). Among them, 21 upregulated differentially expressed genes and 78 downregulated differentially expressed genes were common to all datasets. We used these genes to perform KEGG and GO analyses. GO analysis indicated that the GO terms notably enriched in the differentially expressed genes were “single-multicellular organism process,” “single-organism process,” “single-organism developmental process,” and “developmental process” (Table 2). KEGG analysis suggested that the pathways notably enriched in the differentially expressed genes were “galactose metabolism,” “EGFR tyrosine kinase inhibitor resistance,” “pathways in cancer,” and “PI3K-Akt pathway” (Table 2). We noticed that one of the significantly enriched GO terms was “serotonin uptake” and that GPM6B was involved with this GO term. According to the related literature, GPM6B plays a biological role in various tumors, but there has been no research on GPM6B in PCa. Therefore, we decided to conduct further research on GPM6B.To determine the expression pattern of GPM6B in PCa, we further analyzed GSE46602 (*t* = 5.072, *p* < 0.0001), GSE69223 (*t* = 7.522, *p* < 0.0001), GSE27616 (*t* = 3.174, *p* = 0.0088), and a TCGA cohort (*t* = 11.52, *p* < 0.0001). As shown in Figures 1(c)–1(f), GPM6B expression was significantly decreased in PCa tissues compared to that in normal prostate tissues.

### 3.2. The Expression of GPM6B Is Negatively Correlated with the Clinical Stage of PCa

**Figure 2:**
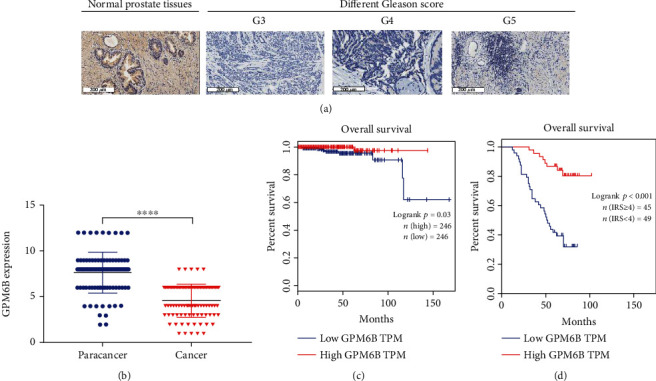
The expression of GPM6B is negatively correlated with the clinical stage of prostate cancer. (a) Immunohistochemical staining of surgical specimens from 94 prostate cancer patients was performed. (b) Statistical analysis of the immunohistochemistry results. (c) Kaplan-Meier survival curves and the log-rank test were used to assess the effect of GPM6B expression on survival in patients with PCa using data from the TCGA database. (d) The effect of GPM6B expression on survival in patients with PCa was determined using data from the First Affiliated Hospital of Zhengzhou University.

We collected clinical-pathological data and paraffin-embedded tissue specimens from patients with PCa. A total of 94 PCa patients with complete clinical and pathological information and pathological specimens were enrolled. To further analyze the data, the patients were divided into two groups according to the median clinical and pathological parameters. Patient clinical data are shown in Table 3. Next, we performed immune-histochemical (IHC) staining and scored the surgical specimens from 94 PCa patients. GPM6B was mainly observed in the cell membrane. Moreover, GPM6B was significantly downregulated in PCa tissues compared to normal prostate tissues (Figures 2(a) and 2(b)). According to the IHC scores of the tumor tissues, the study cohort was divided into two groups; 49 patients made up the low GPM6B expression group (IRS < 4), and 45 patients made up the high GPM6B expression group (IRS ≥ 4). Chi-square test was used to judge the association between GPM6B expression and clinic pathological parameters. The GPM6Bexpression was notably related to clinical stage (*χ*^2^ = 7.318, *p* = 0.007), T classification (*χ*^2^ = 5.372, *p* = 0.020), lymph node metastasis (*χ*^2^ = 5.556, *p* = 0.018), and distant metastasis (*χ*^2^ = 4.765, *p* = 0.029), but was not significantly related to age (*χ*^2^ = 1.514, *p* = 0.029) and Gleason score (*χ*^2^ = 2.559, *p* = 0.110) (Table 4). We then investigated whether GPM6B dysregulation is related to the prognosis of 94 patients. Follow-up of patients in the study cohort, 40 patients were observed to have died, and 54 patients were still alive. Kaplan-Meier survival curves and the log-rank test were used to estimate the effect of GPM6B expression on the survival rates of patients with PCa. First, data from a TCGA database were analyzed. High GPM6B expression was found to be a favorable prognostic predictor for OS of PCa patients in a TCGA cohort (Figure 2(c)). In addition, we analyzed data from the research cohort consisting of 94 patients. The results were consistent with those of the analysis of data from the TCGA database (Figure 2(d)). Furthermore, we used the Cox proportional hazards model for univariate and multivariate analyses to analyze the correlation between GPM6B expression with clinic pathological features and patient outcome. In univariate analysis, GPM6B expression, clinical stage, Gleason score, T classification, lymph node metastasis, and distant metastasis were significantly related to OS (Table 5). In multivariate Cox regression analysis, Gleason score, distant metastasis, and GPM6B expression were independent predictors of prognosis (Table 5).

### 3.3. GPM6B Inhibits PCa Cell Proliferation

**Figure 3:**
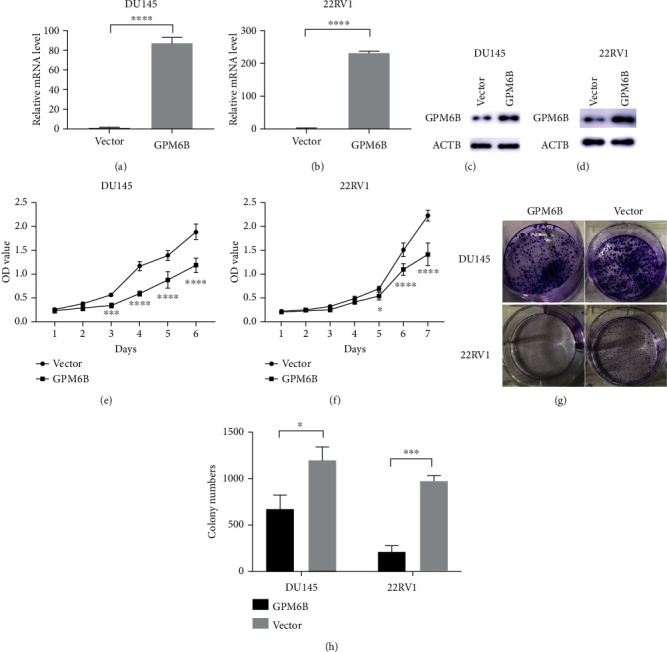
GPM6B inhibits PCa cell proliferation. (a–d) The transfection efficacy of the GPM6B plasmid was confirmed by qPCR and Western blotting. (e–h) The proliferation of DU145 and 22RV1 cells transfected with GPM6B-plasmid was detected by CCK8 assay and colony formation assay.

To investigate the role of GPM6B in PCa, we constructed LV-GPM6B-3flg-GFP-Puro overexpression plasmid, which was then transfected with the GPM6B plasmid into DU145 and 22RV1 cells. The transfection efficacy was confirmed using qPCR and Western blot analyses (Figures 3(a)–3(d)). After GPM6B plasmid transfection, we detected cell proliferation by CCK8 assay. PCa cell proliferation was inhibited by GPM6B overexpression (Figures 3(e) and 3(f)), and this inhibition became more significant over time (*p* < 0.001). In addition, the colony formation ability of GPM6B-overexpressing PCa cell lines was significantly reduced (Figures 3(g) and 3(h)).

### 3.4. GPM6B Reduces Serotonin Intake to Inhibit the PCa Cell Growth

**Figure 4:**
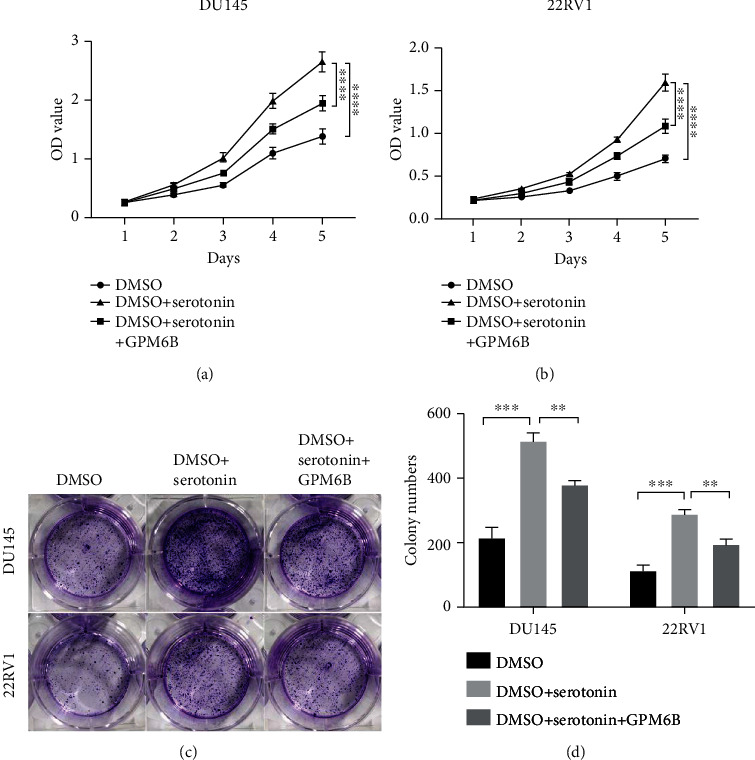
GPM6B reduces serotonin intake to inhibit the PCa cell growth. (a, b) Detect the effect of serotonin and GPM6B expression on the proliferation of prostate cancer cell lines by cck8 assay. (c, d) Detect the effect of serotonin and GPM6B expression on the proliferation of prostate cancer cell lines by colony formation assay.

GPM6Bcan serve as a serotonin intake inhibitor in many other cancers, and we are curious about its function in PCa cells. So, we further investigated the influence of serotonin on the proliferation of PCa. In CCK8 assays, serotonin was used to treat PCa cell lines and significantly promoted the proliferation of these PCa cell lines. When GPM6B was overexpressed, it could reverse the proliferation effect of serotonin on the PCa cell line (Figures 4(a) and 4(b)). The results of the colony formation assay also shows serotonin can promote the PCa cells growths but overexpressing GPM6B can reverse its effect (Figures 4(c) and 4(d)), and this is consistent with the results of the CCK8 assay.

### 3.5. GPM6B Reduces Serotonin Intake by Competing with SERT

**Figure 5:**
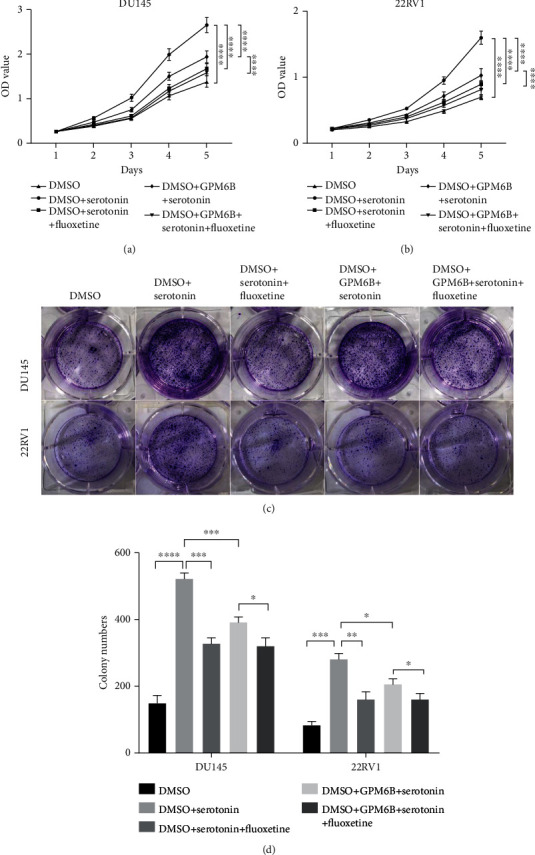
GPM6B reduces serotonin intake by competing with SERT. (a, b) Detect the effect of fluoxetine and GPM6B expression on the proliferation of prostate cancer cell lines by cck8 assay. (c, d) Detect the effect of fluoxetine and GPM6B expression on the proliferation of prostate cancer cell lines by colony formation assay.

Related studies have confirmed that GPM6B physically binds the serotonin transporter (SERT) in the cell membrane, which significantly reduces the uptake of serotonin. To determine whether the proliferative effect of serotonin on the PCa cell lines was due to SERT, we used fluoxetine (a SERT antagonist) to treat PCa cell lines to which serotonin had been added. In CCK8 assays, fluoxetine treatment significantly inhibited the proliferation of PCa cell lines compared to cells without fluoxetine treatment. Fluoxetine treatment can reverse the proliferation effect of serotonin, which is similar to the effect played by GPM6B (Figures 5(a) and 5(b)). The combination of fluoxetine and ectopically expressed GPM6Bcan has the same effect with individual, which means they are working on the same pathway. The results of the colony formation assay also show the fluoxetine treatment and ectopically expressed GPM6B have the same effect on PCa cell growth, which is consistent with the results of the CCK8 assay (Figures 4(c) and 4(d)). In conclusion, the GPM6B can target SERT to inhibit PCa cells growth by reducing the serotonin intake of PCa cells.

## 4. Discussion

In the present research, the GPM6B expression level was analyzed in 94 PCa patients and confirms its negative relationship with PCa clinic pathological. We validated the expression pattern of GPM6B in PCa and verified for the first time that differential expression of GPM6B is associated with the survival status of patients with PCa. The following experimental results support this view: (1) GPM6B was lower in PCa tissues than in normal prostate tissues, as revealed by analysis of three independent GEO datasets and a TCGA cohort; (2) IHC analysis of PCa tissues and adjacent normal tissues suggested that GPM6B expression in tumor tissues is reduced; (3) the expression of GPM6B in tumor tissues was significantly related to clinical stage, T classification, lymph node metastasis, and distant metastasis, but was not significantly related to age and Gleason score; (4) low GPM6B expression in PCa was found to be related to worse survival. These data suggest the usefulness of GPM6B for the prognostic assessment of PCa.

GPM6B is a transmembrane protein that belongs to the proteolipid protein family. Recent studies have indicated that GPM6B plays an important role in myelination, neuronal differentiation, and osteoblast differentiation. Some oncology research data have also indicated that GPM6B can be used as a biomarker for gynecological malignancies and breast carcinoma [16, 18]. Studies by Fjorback et al. [14] confirmed that the expression of Gpm6B significantly decreased serotonin uptake. Also, PCa cell proliferation is reported and can be inhibited by serotonin receptor antagonists [19]. Furthermore, high doses of serotonin are speculated to directly promote tumor cell mitosis [19].

In our experiments, serotonin could promote the proliferation of PCa cell lines, which is consistent with a previous study [14, 15, 19]. On the other side, the overexpression of GPM6B can reverse the effect of serotonin in PCa cell growth. This means GPM6B may inhibit the proliferation of PCa cell lines by affecting the intake of serotonin. The combination of fluoxetine with the overexpression of GPM6B can no longer slow down the growth rate of PC3 and 22Rv1 cells when compared with fluoxetine alone, which means GPM6B and fluoxetine are working in the same pathway.

In the Kaplan-Meier survival analysis of the TCGA cohort, only 10 of the 492 patients died (2.03%), while in our study cohort, 40 of the 94 patients died (42.5%). The number of patient deaths in our cohort was significantly higher than the TCGA cohort. This is because the patients included in the study cohort were patients with prostate cancer who were seen in our hospital from 2011 to 2013. Due to the implementation of tiered diagnosis and treatment, most of the patients with prostate cancer admitted to our hospital are more serious patients. Additionally, at the time of this study, early screening for prostate cancer was not as common as it is currently. Therefore, most of the patients included in the study cohort were elderly patients and patients with clinical stage III or IV PCa (Table 3). Observing more end events can make Kaplan-Meier survival analysis results more accurate, which is one of the advantages of our research cohort.

There are limitations to our research. First, the reasons for the differential expression of GPM6B in prostate cancer tissues and normal tissues adjacent to the cancer were not investigated. Second, there was no further verification of the mechanism of action of GPM6B. Third, the sample size was relatively small. We will further explore the mechanism of GPM6B's role in prostate cancer in follow-up research and expand the sample size. Our study clarified for the first time that GPM6B in PCa is downregulated and associated with the prognosis. GPM6B may play the role of tumor suppressor genes in PCa through mediated changes in serotonin uptake. These results suggested the potential usefulness of GPM6B for the prognostic assessment of PCa and provide clues for the development of new targets for the treatment of PCa.

## Figures and Tables

**Table 1:** The composition of the GEO dataset.

	GSE46602	GSE69223	GSE27616
PCa	36	15	9
Normal prostate	14	15	4

**Table 2:** Results of gene ontology (GO) categories and Kyoto Encyclopedia of Genes and Genomes (KEGG) pathway analysis.

Category	Description	Gene counts	*p* value
Upregulated gene			
GO:0044767	Single-organism developmental process	14	5.37*E*-08
GO:0032502	Developmental process	14	6.59*E*-08
GO:0050794	Regulation of cellular process	17	1.31*E*-07
GO:0048468	Cell development	9	2.01*E*-07
GO:0048731	System development	12	2.33*E*-07
GO:0050789	Regulation of biological process	17	2.88*E*-07
GO:0048856	Anatomical structure development	13	3.10*E*-07
GO:0045595	Regulation of cell differentiation	8	5.05*E*-07
GO:0009987	Cellular process	19	5.14*E*-07
GO:0044699	Single-organism process	18	5.66*E*-07
hsa00524	Butirosin and neomycin biosynthesis	1	0.0031634
hsa00120	Primary bile acid biosynthesis	1	0.0094615
hsa00052	Galactose metabolism	1	0.0167615
hsa00051	Fructose and mannose metabolism	1	0.0178001
hsa05033	Nicotine addiction	1	0.0214272
Downregulated gene			
GO:0044699	Single-organism process	60	5.32*E*-16
GO:0044707	Single-multicellular organism process	41	5.59*E*-15
GO:0032501	Multicellular organismal process	43	3.15*E*-14
GO:0044763	Single-organism cellular process	55	3.92*E*-14
GO:0048513	Animal organ development	30	4.57*E*-14
GO:0005488	Binding	60	5.10*E*-14
GO:0009653	Anatomical structure morphogenesis	27	1.27*E*-13
GO:0044421	Extracellular region part	32	1.54*E*-13
GO:0044767	Single-organism developmental process	38	3.65*E*-13
GO:0051610	Serotonin uptake	1	0.011691
hsa01521	EGFR tyrosine kinase inhibitor resistance	4	2.44*E*-05
hsa04360	Axon guidance	5	2.96*E*-05
hsa04510	Focal adhesion	5	5.73*E*-05
hsa05200	Pathways in cancer	6	0.0001435
hsa04151	PI3K-Akt signaling pathway	5	0.0006111

**Table 3:** Patient characteristics (*n* = 94).

Characteristics	Values
Age (years)	71 ± 8.78
Clinical stage (I/II/III/IV)	22/9/34/29
Gleason score (<7/≥7)	36/58
T classification (T1/T2/T3/T4)	8/54/3/29
Lymph node metastasis (present/absent)	15/79
Distant metastasis (present vs. absent)	29/65
GPM6B staining (IRS < 4/IRS ≥ 4)	49/45

**Table 4:** Relationship between the expression of GPM6B in prostate cancer and clinicopathological parameters.

Characteristics	*N*	GPM6B IRS ≥ 4	GPM6B IRS < 4	*χ* ^2^	*p* value
Age					
≥70	48	25	21	1.514	0.219
<70	46	20	28		
Clinical stage					
III, IV	63	24	39	7.318	0.007
I, II	31	21	10		
Gleason score					
≥7	58	24	34	2.559	0.110
<7	36	21	15		
T classification					
T3, T4	32	10	22	5.372	0.020
T1, T2	62	35	27		
Lymph node metastasis					
Present	15	3	12	5.556	0.018
Absent	79	42	37		
Distant metastasis					
Present	29	9	20	4.765	0.029
Absent	65	36	29		

**Table 5:** Analysis of different prognostic factors in 94 PCa patients.

Prognostic parameter	Univariate analysis			Multivariate analysis		
	HR	95% CI	*p* value	HR	95% CI	*p* value
Expression of GPM6B (IRS < 4/IRS ≥ 4)	0.180	0.083-0.392	0.001	0.207	0.094-0.456	0.001
Age (≥70 vs. <70)	1.278	0.685-2.384	0.440			n.s.
Clinical stage (III-IV vs. I-II)	4.979	1.941-12.773	0.001			n.s.
Gleason score (≥7 vs. <7)	3.535	1.621-7.706	0.001	2.992	1.344-6.663	0.007
T classification (T3, T4 vs. T1, T2)	3.384	1.801-6.358	0.001	2.278	1.175-4.413	0.015
Lymph node metastasis (present vs. absent)	2.931	1.455-5.903	0.003			n.s.
Distant metastasis (present vs. absent)	3.340	1.781-6.263	0.001			n.s.

n.s.: nonsignificant.

## Data Availability

All data generated or analyzed during this study are included either in this article or in the supplementary information files.
